# The reliability and validity of the Turkish version of the multiple sclerosis impact scale-29

**DOI:** 10.55730/1300-0144.5426

**Published:** 2022-06-18

**Authors:** Fatih ÖZDEN, Mehmet ÖZKESKİN, Nur YÜCEYAR

**Affiliations:** 1 Department of Health Care Services, Köyceğiz Vocational School of Health Services, Muğla Sıtkı Koçman University, Muğla, Turkey; 2 Department of Physiotherapy and Rehabilitation, Faculty of Health Sciences, Ege University, İzmir, Turkey; 3 Department of Neurology, Faculty of Medicine, Ege University, İzmir, Turkey

**Keywords:** MSIS-29, sypmtom severity, patient reported outcomes, psychometrics

## Abstract

**Background/aim:**

The purpose of the study was to cross-culturally adapt the Multiple Sclerosis Impact Scale-29 (MSIS-29) into Turkish and evaluate its reliability and validity in patients with Multiple Sclerosis (MS).

**Materials and methods:**

A total of 119 individuals with MS were enrolled in the research. The neurologist classified the patients with Expanded Disability Status Scale (EDSS). In the initial evaluation, patients completed the Multiple Sclerosis Impact Scale-29 (MSIS-29), the Multiple Sclerosis International Quality of Life (MusiQoL), EuroQol-5D-3L (EQ-5D-3L), and Beck Depression Scale (BDS), respectively one week later, the MSIS-29 evaluation was repeated. Internal consistency, test-retest reliability, and construct validity were assessed, separately.

**Results:**

The mean age of the total sample was 38.2 ± 10.6 years. The test-retest reliability of both subscores of the MSIS-29 was excellent (>0.80). Internal consistency of the MSIS-29 physical and psychological score was 0.968 and 0.914, respectively. Both of the subscores had excellent internal consistency (>0.80). There was a strong relationship between MSIS-29 physical score with MusiQoL, EQ-5D-3L (index), EQ-5D-3L (VAS), and BDS scores (p < 0.01, r > 0.50). MSIS-29 physical was moderately related to EDSS (p < 0.01, r = 0.381). MSIS-29 psychological score was strongly correlated with MusiQoL, EQ-5D-3L (index), EQ-5D-3L (VAS), and BDS scores (p < 0.01, r > 0.50). On the other hand, there was a weak correlation between MSIS-29 psychological score and EDSS (p < 0.01, r = 0.300).

**Conclusion:**

Turkish version of the MSIS-29 is a reliable and valid tool in individuals with MS.

## 1. Introduction

Multiple Sclerosis (MS) negatively affects individuals’ physical/psychological functions and health-related quality of life [1]. The health-related quality of life of individuals with MS is lower than individuals with other chronic diseases [2,3]. In addition to the treatments developed for MS and related conditions, clinical evaluation methods that reveal the effects of MS on individuals in daily life and community-based rehabilitation methods are needed [4]. The Expanded Disability Status Scale (EDSS) [5] and Multiple Sclerosis Functional Composite (MSFC) [6] are primarily preferred in the assessment of disability in patients with MS. However, among the evaluation methods, patient-reported outcomes (PROs) are used to evaluate the individual’s health status with MS from the individual’s perspective owing to their direct answers [7, 8]. PROs show the effect of the disease on the individual more broadly [4]. Questionnaires could be performed in any environment quickly and easily [9]. PROs include symptoms, daily living activities, quality of life, patient satisfaction and compliance to reveal the patient’s comprehensive clinical status [10,11]. An appropriate PRO can detect changes in the ability of an individual with MS to perform activities of daily living [12]. There are MS-specific and non-MS-specific PROs used in individuals with MS [13]. Among those specific to MS, SymptomScreen [14] points out MS symptoms, Multiple Sclerosis International Quality of Life (MusiQoL) exposes the health-related quality of life [15], and Multiple Sclerosis Impact Scale-29 (MSIS-29) represents the physical and psychological quality of life [16]. It was found that MSIS-29 was valid and reliable on hospital-based samples (MS patients receiving corticosteroids, MS patients during rehabilitation, and also primary progressive MS patients) and was identical to community samples [17]. The physical subdimension of MSIS-29 is one of the first scales [16] developed explicitly for MS, and has also been shown to be correlated with the commonly used EDSS, MSFC, and Guy’s Neurological Disability Scale (GNDS) [18]. Validity and reliability studies of MSIS-29 were conducted in English (original version) [17], Norwegian [19], Polish [20], Korean [21], Finnish [4], and Croatian [22]. The present research aimed to cross-culturally adapt the Multiple Sclerosis Impact Scale-29 (MSIS-29) into Turkish and evaluate its reliability and validity in patients with MS.

## 2. Materials and methods

### 2.1. Translation and adaptation process

The original MSIS-29 developer granted the authorization for the translation, adaptation, reliability, and validity analysis of the Turkish MSIS-29. The common procedures of Beaton et al. and Guillemin et al. were used for the translation and adaptation stages [23, 24]. The Turkish version of the MSIS-29 is presented in Appendix 1.

### 2.2. Sample size estimation

The sample size of the research was conducted with the expected Cronbach’s alpha (H_1_) of approximately 0.80 (based on the original development study and other versions), the minimum acceptable Cronbach’s alpha (H_0_) of 0.70, the significance level of 0.05, power of 0.80, number of items of the MSIS-29, and 15% drop-out rate. Accordingly, it was determined that at least 119 cases should be evaluated to carry out the study [25]. On the other hand, in calculating test-retest reliability, 0.60 for minimum acceptable reliability (ICC_0_), 0.85 for expected reliability (ICC_1_), significance level of 0.05, power of 0.80, 2 repetitions per subject (k), and 15% drop-out rate were considered [26]. Finally, 31 cases were decided to be sufficient for the reproducibility analysis. As a result, 119 individuals for the first test and 36 for the retest were enrolled in the research.

### 2.3. Study design

A psychometric analysis study was conducted at Ege University, Neurology Department. One hundred nineteen individuals with MS were enrolled in the study. The study protocol was approved by the ethics committee of Ege University (No: 21-5T/97). Mc Donald criteria were used for the diagnosis of MS. Turkish-speaking patients over 18 years old were included in the study. Exclusion criteria of the research were; (1) no relapse history for one month, (2) EDSS score > 7.5 (2), being bedridden (3), other conditions that alter mobility and function, (4) cognitive impairments.

Since our study included cultural adaptation, demographic and socio-cultural characteristics of the patients were recorded. Then, the physical and individual characteristics of the participants were documented. Our patients were evaluated twice at a one-week interval due to the test-retest reliability study method. In the initial assessment, patients completed the Multiple Sclerosis Impact Scale-29 (MSIS-29) [16], The Multiple Sclerosis International Quality of Life (MusiQoL) [27], EuroQol-5D-3L (EQ-5D-3L) [28], and Beck Depression Scale (BDS) [29], respectively. The clinical neurologist evaluated the patients and fulfilled the Expanded Disability Status Scale (EDSS) [5] scores. In the second evaluation conducted one week later, 36 randomly selected MS patients filled MSIS-29 again. Standardized, reliable, and valid Turkish versions of all questionnaires were used.

#### Multiple Sclerosis Impact Scale-29 (MSIS-29)

Developed by Hobart et al. in 2001 [16]. The tool includes a 20-statements containing the physical parameters related to MS disease and a 9-item covering psychological problems. The physical part includes items 1 to 20. The psychological part is ranged from items 21 to 29. Participants are asked to respond to each item regarding the condition’s impact on their daily life in the last two weeks [30]. The patients select the answer that strongly represents their status and responds on a 5-point Likert scale for every item. The patient’s scores on the two subscales could be summed and converted to a measure between 0 to 100. High scores show a high disease impact [22].

#### The Multiple Sclerosis International Quality of Life (MusiQoL)

Validity and reliability studies of MusiQoL scales in the Turkish population were conducted by Idiman et al. [15].

#### EuroQol-5D-3L (EQ-5D-3L)

EQ-5D-3L includes two subscales: the quality-based index system and the visual analog scale (VAS). Turkish validation has been demonstrated [28].

#### Beck Depression Scale (BDS)

Kapci et al. carried out a reliability and validity study of the Turkish BDS. This tool represents the depression severity of the patients [29].

#### Expanded Disability Status Scale (EDSS)

Kurtzke developed EDSS in 1983 to evaluate disability, and it is widely used to evaluate MS patients. EDSS score is calculated by evaluating pyramidal, brainstem, cerebellar, visual, and sensory systems, and the intestinal-bladder and mental functions [5].

### 2.4. Statistical analysis

Statistical analysis was calculated with SPSS for Windows v25.0 (SPSS Inc, Chicago, IL, USA) software. The mean and standard deviation were presented for the quantitative variables. Percentage distribution is presented for qualitative data. The homogeneity of the participants was calculated with the Shapiro-Wilk test. The confidence interval (CI) for Intraclass Correlation Coefficient (ICC) and correlational analysis was accepted as 0.95.

#### 2.4.1. Reliability

Two main analyzes were performed for reliability. Firstly, Cronbach’s alpha value was calculated for each item of the questionnaire and the total score in order to evaluate whether the 29 items of MSIS-29 were consistent with each other. A score of alpha values >0.80 was considered excellent for internal consistency [31]. Secondly, test-retest reliability was evaluated. For the reproducibility of the MSIS-29, the similarity between the two separate assessments one week apart was observed with the Intraclass Correlation Coefficient (ICC, 95% CI). This measurement investigated whether the Turkish version of MSIS-29 gave similar results in different measurements. The Shrout-Fleiss (2,1) type ICC model was preferred. An ICC value above 0.80 is considered perfectly reliable [32].

#### 2.4.2. Validity

Construct validity of MSIS-29 was evaluated with Spearman or Pearson correlation coefficient regarding the normal distribution of the relevant parameter. MSIS-29 was compared with MusiQoL, EQ-5D-3L, and BDS. High correlation indicates high construct validity under convergent validity. If the r-value > 0.5, the validity was interpreted as strong. 0.35 < r > 0.50 was considered moderate and weak if the value <0.35 [33].

## 3. Results

A total of 119 individuals with MS (91 women, 27 men) were enrolled in the research. The mean age of the total sample was 38.2 ± 10.6 years. A vast majority of the patients were educated in a university or higher degree (64.6%). The mean MS disease duration of the individuals was 15.5 ± 3.8 years. The other individual characteristics of the patients related to cross-cultural adaptation are given in Table 1. In addition, the mean scores of the clinical assessments are presented in Table 2. The patients did not report any difficulties with the Turkish version of the MSIS-29 in terms of comprehensibility.

### 3.1. Reliability

The test-retest reliability of both subscores of the MSIS-29 was excellent. The ICC score of the MSIS-29 physical subscale and MSIS-29 phycological subscale was 0.938 (CI: 0.87–0.96) and 0.939 (CI: 0.88–0.96), respectively. Internal consistency of the MSIS-29 physical and psychological score was 0.968 and 0.914, respectively. Both of the subscores had excellent internal consistency. Besides, all items’ alpha value was excellent (>0.80) (Table 3).

### 3.2. Validity

There was a strong relationship between MSIS-29 physical score with MusiQoL, EQ-5D-3L (index), EQ-5D-3L (VAS), and BDS scores (p < 0.01, r > 0.50). MSIS-29 physical was moderately related to EDSS (p < 0.01, r = 0.381). MSIS-29 psychological score was strongly correlated with MusiQoL, EQ-5D-3L (index), EQ-5D-3L (VAS), and BDS scores (p < 0.01, r > 0.50). On the other hand, there was a weak correlation between MSIS-29 psychological score and EDSS (p < 0.01, r = 0.300) (Table 4).

## 4. Discussion

The present study investigated the psychometric properties of the Turkish version of the MSIS-29. MSIS-29 is one of the most widely and effectively used questionnaires in evaluating patients with multiple sclerosis [16,30]. Focusing on both the physiological and psychological dimensions of the effects of the disease on patients, the MSIS-29 questionnaire is a comprehensive evaluation PRO. Since it is comprehended that the questionnaires should be standardized and adapted for the relevant language, a Turkish standardized version would significantly contribute to a clinical evaluation in the rehabilitation process for individuals living in Turkey and Europe whose native language is Turkish [34]. MSIS-29 has been used in the UK’s Web Portal of the “UK MS Register” and proxy use in another study [35,36]. The psychometric properties have not been demonstrated for Turkish. According to the results, the internal consistency of the Turkish MSIS-29 was high, the test-retest reliability was excellent, and the construct validity was sufficient.

The ICC value of the physical and psychological subscores of the Turkish version of MSIS-29 was above 0.80. Both subscores were highly reliable. The ICC value found in the development study (physical: 0.81, psychological: 0.78) [16], the Korean version (physical: 0.90, psychological: 0.78) [21], the Norwegian version (physical: 0.92, psychological: 0.85) [19] is largely identical to the values of our study. In this respect, MSIS-29 can reliably fulfill the same clinical situation in different measurements. Our psychological subscore had a higher ICC value (>0.9) than the other versions. Since the BDS mean of our sample was as low as about 10, it can be deduced that the effect of illness related to depression may be relatively low. Therefore, we concluded that the neuropsychiatric changes, which did not become a complicated situation, could be more clearly expressed by the patients.

The Turkish version of the MSIS-29’s Cronbach’s alpha value for physical and psychological subscores was excellent (>0.80). In the development study, Cronbach’s alpha values in the original development study (physical: 0.96, psychological: 0.91) [16], in the Croatian version (physical: 0.95, psychological: 0.93) [22], in the Finnish version (physical: 0.97, psychological: 0.90) [4], in the Norwegian version (physical: 0.88, psychological: 0.97) [19], and in the Korean version (physical: 0.97, psychological: 0.96) [21] were high. Mostly, alpha values are seen to be above 0.90. These scores pointed out that physical and psychological subscores can be evaluated consistently in the relevant clinical group of individuals. In other words, the 20-item physical subscale items are consistent with each other, representing the physical subtotal score, while the 9-item psychological subscore is consistent with each other to represent the neuropsychiatric condition.

For construct validity, we used popular and gold-standard questionnaires in the field such as MusiQoL, EQ-5D-3L (index), EQ-5D-3L (VAS), BDS, and EDSS. MusiQoL reveals the MS-based quality of life and the impact of disease symptoms on life, EQ-5D-3L indicates the general quality of life, BDS shows psychological state, and EDSS demonstrates the individuals’ disability due to MS. Turkish MSIS-29 was highly correlated with all PROs (r > 0.50). However, the physical and psychological subdimension of the Turkish MSIS-29 was moderately and lowly correlated with the EDSS (r > 0.35, r < 0.35). EDSS is a clinician-based objective criterion. Therefore, our EDSS results were relatively low than other PROs comparisons. In the development study, correlations with SF-36, EQ-5D, FAMS, GHQ-12, Barthel Index were examined (r = 0.05; −0.88) [16]. Similar to our study, there were different levels of similarity from low to high. In the Croatian validation study, a correlation coefficient between 0.35 and 0.66 was obtained with DASS-21 [22]. They observed a lower correlation than the BDS results we used similarly. The observed correlation coefficient with EDSS, EQ-5D, and FSS in the Finnish version ranged from 0.2 to 0.8 [4]. These results confirmed our construct validity. Finally, the correlation of MSIS-29 with EDSS, FSS, PHQ, and MusiQoL in the Korean version was examined [21]. Construct validity results of our study showed similarity with the Korean study, in which a correlation was observed at levels ranging from −0.01 to 0.87.

### 4.1. Limitations

Some limitations of the study should be acknowledged and explained. First, according to the COSMIN declaration, responsiveness analysis, which is one of the essential measurements, was not performed for psychometric analysis in our study [37]. Because this analysis requires a method that requires long-term follow-up of patients or their response to treatment. Second, instead of retesting the entire sample, 36 people randomly determined by sample size calculation were retested. We preferred this pragmatic approach, especially when it was difficult to reach all patients. However, further studies may perform the reproducibility analysis with a larger sample.

### 4.2. Conclusions

The results of our study revealed that the Turkish version of MSIS-29 was translated with a culturally appropriate adaptation process. According to the results of psychometric analysis, the MSIS-29 Turkish version is a valid, reliable PRO tool. Owing to MSIS-29, clinicians could specifically assess the disease impact of individuals with MS, both physiologically and psychologically.

## Supplementary Information



## Figures and Tables

**Table 1:** The participants’ individual characteristics.

n: 119	Total
Age (years, mean ± SD)	38.2 ± 10.6
BMI	24.0 ± 3.8
Gender (n, %)	
Women	91 (76.5)
Men	27 (23.5)
Duration of MS condition (years, mean ± SD)	15.5 ± 3.8
Education status (n, %)	
Elementary school	14 (11.8)
Secondary school	4 (3.4)
Senior high school	24 (20.2)
Bachelors or postgraduate	77 (64.6)
Marital status (n, %)	
Married	75 (63.0)
Single	44 (37.0)
Employment (n, %)	
Yes	53 (44.5)
No	66 (55.5)

SD: standard deviation, n: number of patients, BMI: body mass index

**Table 2:** Mean scores of the assessments.

n: 119	Mean±SD	Range
EDSS	1.7 ± 1.4	(0–7.5)
MSIS-29 (physical)	22.4 ± 21.2	(0–77.5)
MSIS-29 (psychological)	32.7 ± 22.7	(0–83.3)
MusiQoL	44.8 ± 12.9	(21.9–78.0)
EQ-5D-3L (index)	0.7 ± 0.1	(0.1–1)
EQ-5D-3L (VAS)	75.6 ± 18.3	(30–100)
BDS	10.8 ± 8.6	(0–34)

SD: standard deviation, n: number of patients

**Table 3:** The internal consistency and test-retest reliability of the MSIS-29 (physical).

	Test (Mean ± SD)	Retest (Mean±SD)	ICC (95% CI)	α
**Item 1**	2.1 ± 1.0	2.0 ± 0.8	0.593 (0.20–0.79)	0.967
**Item 2**	1.8 ± 1.0	1.7 ± 0.9	0.599 (0.21–0.79)	0.968
**Item 3**	2.1 ± 1.0	2.1 ± 1.0	0.496 (0.01–0.74)	0.966
**Item 4**	1.9 ± 1.0	2.2 ± 1.1	0.717 (0.44–0.85)	0.965
**Item 5**	1.7 ± 1.0	1.8 ± 0.9	0.710 (0.43–0.85)	0.966
**Item 6**	2.0 ± 0.9	2.0 ± 0.8	0.807 (0.62–0.90)	0.967
**Item 7**	2.0 ± 1.0	1.9 ± 0.9	0.869 (0.74–0.93)	0.966
**Item 8**	2.1 ± 1.1	2.1 ± 0.8	0.845 (0.69–0.92)	0.966
**Item 9**	1.8 ± 0.8	1.8 ± 0.8	0.940 (0.88–0.97)	0.969
**Item 10**	1.9 ± 1.1	2.0 ± 0.9	0.854 (0.71–0.92)	0.966
**Item 11**	1.7 ± 1.1	1.6 ± 0.9	0.943 (0.88–0.97)	0.965
**Item 12**	1.4 ± 0.9	1.4 ± 0.7	0.909 (0.82–0.95)	0.966
**Item 13**	1.6 ± 0.9	1.6 ± 0.9	0.948 (0.89–0.97)	0.965
**Item 14**	1.8 ± 1.2	1.9 ± 1.1	0.952 (0.90–0.97)	0.965
**Item 15**	1.6 ± 1.0	1.7 ± 1.0	0.937 (0.87–0.96)	0.966
**Item 16**	1.8 ± 1.1	1.8 ± 1.0	0.975 (0.95–0.98)	0.965
**Item 17**	1.6 ± 1.0	1.5 ± 0.8	0.877 (0.75–0.93)	0.966
**Item 18**	2.1 ± 1.2	2.0 ± 1.1	0.773 (0.55–0.88)	0.965
**Item 19**	1.8 ± 1.1	1.7 ± 0.8	0.855 (0.71–0.92)	0.965
**Item 20**	2.3 ± 1.3	2.1 ± 1.1	0.895 (0.79–0.94)	0.968
**MSIS-29 (physical)**	**22.4 ± 21.2**	**22.3 ± 19.4**	**0.938 (0.87**–**0.96)**	**0.968**
**Item 21**	2.1 ± 1.0	2.1 ± 1.0	0.711 (0.43–0.85)	0.927
**Item 22**	2.2 ± 1.3	2.0 ± 0.8	0.861 (0.72–0.92)	0.905
**Item 23**	2.4 ± 1.2	2.2 ± 0.9	0.865 (0.73–0.93)	0.895
**Item 24**	2.3 ± 1.0	2.1 ± 0.8	0.871 (0.74–0.93)	0.909
**Item 25**	2.3 ± 1.1	2.0 ± 0.9	0.815 (0.63–0.90)	0.894
**Item 26**	2.5 ± 1.1	2.3 ± 0.9	0.792 (0.59–0.89)	0.900
**Item 27**	2.3 ± 1.1	1.9 ± 0.9	0.881 (0.76–0.94)	0.904
**Item 28**	2.1 ± 1.1	2.0 ± 0.7	0.771 (0.55–0.88)	0.902
**Item 29**	2.1 ± 1.2	2.1 ± 1.0	0.812 (0.63–0.90)	0.899
**MSIS-29 (psychological)**	**32.7 ± 22.7**	**28.0 ± 17.3**	**0.939 (0.88**–**0.96)**	**0.914**

n: number of patients, ICC: intra-class correlation coefficient, CI: confidence interval, α: Cronbach’s alpha

**Table 4:** Construct validity of the MSIS-29.

n: 119	MSIS-29 (physical)	MSIS-29 (psychological)
**MusiQoL**	0.774*	0.853*
**EQ-5D-3L (index)**	−0.786*	−0.749*
**EQ-5D-3L (VAS)**	−0.832*	−0.755*
**BDS**	0.542*	0.717*
**EDSS**	0.381*	0.300*

* p < 0.01
