# Supplementary material for: The reliability and validity of the Turkish version of the multiple sclerosis impact scale-29
Source: Turk J Med Sci. 2022 Jun 18;52(4):1216–22. doi: 10.55730/1300-0144.5426 (PMC10388122; doi:10.55730/1300-0144.5426)
Supplement: Supplementary file 1 [file SAG-2110-180_1_Appendix_1.docx]

**Multiple Skleroz Etki Skalası (TR-MSIS-29)**

Aşağıdaki ifadeler, Multipl Skleroz’un (MS) son iki haftada, günlük yaşamınız üzerindeki etkisi hakkında görüşlerinizi sorgulamaktadır. Lütfen her ifade için durumunuzu en iyi tanımlayan sayıyı daire içine alın. Lütfen tüm soruları cevaplayın.

| Son iki hafta içinde MS, …………. becerinizi ne kadar kısıtladı. | | Hiç | Biraz | Orta derecede | Oldukça | Aşırı |
| --- | --- | --- | --- | --- | --- | --- |
| 1. | fiziksel olarak zorlu görevler yapma | 1 | 2 | 3 | 4 | 5 |
| 2. | objeleri sıkıca kavrama (ör: musluk açmak) | 1 | 2 | 3 | 4 | 5 |
| 3. | bir şeyler taşıma | 1 | 2 | 3 | 4 | 5 |
| Son iki hafta içinde, …………. sizi ne kadar rahatsız etti? | | Hiç | Biraz | Orta derecede | Oldukça | Aşırı |
| 4. | dengenizle ilgili sorunlar | 1 | 2 | 3 | 4 | 5 |
| 5. | kapalı mekanlarda hareket etme güçlüğü | 1 | 2 | 3 | 4 | 5 |
| 6. | sakar olmak | 1 | 2 | 3 | 4 | 5 |
| 7. | kas sertliği | 1 | 2 | 3 | 4 | 5 |
| 8. | kollarınızdaki ve/veya bacaklarınızdaki ağırlık | 1 | 2 | 3 | 4 | 5 |
| 9. | kollarınızda veya bacaklarınızda titreme | 1 | 2 | 3 | 4 | 5 |
| 10. | kol veya bacaklarınızdaki spazmlar | 1 | 2 | 3 | 4 | 5 |
| 11. | vücudunuzun yapmak istediğiniz bir şeyi yapamaması | 1 | 2 | 3 | 4 | 5 |
| 12. | sizin için bir şeyler yapmalarıyla ilgili başkalarına güvenmek zorunda kalmak | 1 | 2 | 3 | 4 | 5 |
| 13. | evdeki sosyal ve boş zaman aktivitelerinizdeki kısıtlamalar | 1 | 2 | 3 | 4 | 5 |
| 14. | evde, olmak istediğinizden daha fazla sıkışıp kalmak | 1 | 2 | 3 | 4 | 5 |
| 15. | günlük aktivitelerde ellerinizi kullanmada yaşadığınız zorluklar | 1 | 2 | 3 | 4 | 5 |
| 16. | işinize veya diğer günlük aktivitelere harcadığınız zamanı kısaltmak zorunda kalmak | 1 | 2 | 3 | 4 | 5 |
| 17. | ulaşım sorunları (ör: araba, otobüs, tren, taksi vb.) | 1 | 2 | 3 | 4 | 5 |
| 18. | işleri daha uzun sürede yapmak | 1 | 2 | 3 | 4 | 5 |
| 19. | işleri olağan akışında yapmakta güçlük çekmek | 1 | 2 | 3 | 4 | 5 |
| 20. | acilen tuvalete gitme ihtiyacı duymak | 1 | 2 | 3 | 4 | 5 |
| 21. | kendinizi iyi hissetmemek | 1 | 2 | 3 | 4 | 5 |
| 22. | uyku problemleri | 1 | 2 | 3 | 4 | 5 |
| 23. | zihinsel olarak yorgun hissetmeniz | 1 | 2 | 3 | 4 | 5 |
| 24. | MS'inizle ilgili endişeleriniz | 1 | 2 | 3 | 4 | 5 |
| 25. | endişeli veya gergin hissetmek | 1 | 2 | 3 | 4 | 5 |
| 26. | sinirli, sabırsız veya çabuk sinirlenen biri olmak | 1 | 2 | 3 | 4 | 5 |
| 27. | odaklanma problemleri | 1 | 2 | 3 | 4 | 5 |
| 28. | güven eksikliği | 1 | 2 | 3 | 4 | 5 |
| 29. | depresif hissetmek | 1 | 2 | 3 | 4 | 5 |
